# The magnitude of mortality and its predictors among adult patients admitted to the Intensive care unit in Amhara Regional State, Northwest Ethiopia

**DOI:** 10.1038/s41598-023-39190-7

**Published:** 2023-07-25

**Authors:** Tilahun Bizuayehu Demass, Abel Girma Guadie, Tilahun Birara Mengistu, Zenaw Ayele Belay, Amare Alemu Melese, Abraham Amsalu Berneh, Lealem Gedefaw Mihret, Fikirte Estifanose Wagaye, Getasew Mulat Bantie

**Affiliations:** 1 Department of Internal Medicine, School of Medicine, College of Medicine and Health Sciences, University of Gondar, Gondar Town, Ethiopia; 2 Department of Internal Medicine, School of Medicine, College of Medicine and Health Sciences, Bahir Dar University, Bahir Dar City, Ethiopia; 3 Department of Statistics, Injibara University, Injibara Town, Ethiopia; 4 Food Safety, and Microbiology Reference Laboratory, Ethiopian Public Health Institute, Addis Ababa, Ethiopia; 5 Amhara National, Regional State Public Health Institute, Bahir Dar City, Ethiopia

**Keywords:** Health care, Medical research, Signs and symptoms

## Abstract

Despite mortality in intensive care units (ICU) being a global public health problem, it is higher in developing countries, including Ethiopia. However, insufficient evidence is established concerning mortality in the ICU and its predictors. This study aimed to assess the magnitude of ICU mortality and its predictors among patients at Tibebe Ghion specialized hospital, Northwest Ethiopia, 2021. A retrospective cross-sectional study was conducted from February 24th, 2019, to January 24th, 2021. Data were collected from medical records by using pretested structured data retrieval checklist. The collected data was entered into Epi-data version 3.1 and analyzed using R version 4.0 software. Descriptive statistics computed. A simple logistic analysis was run (at 95% CI and *p*-value < 0.05) to identify the determinants for ICU mortality. A total of 568 study participants’ charts were reviewed. The median length of ICU stay was four days. Head trauma and shock were the leading causes of ICU admissions and mortality. The overall mortality rate of the ICU-admitted patients was 29.6% (95% CI: 26%, 33%). Admission in 2020 (AOR = 0.51; 95%CI: 0.31, 0.85), having altered mentation (AOR = 13.44; 95%CI: 5.77, 31.27), mechanical ventilation required at admission (AOR = 4.11; 95%CI: 2.63, 6.43), and stayed < 5 days in the ICU (AOR = 3.74; 95%CI: 2.31, 6.06) were significantly associated with ICU mortality. The magnitude of the ICU mortality rate was moderate. Years of admission, altered mentation, mechanical ventilation required at admission, and days of stay in the ICU were the predictors for ICU mortality. This finding underscores the importance of interventions to reduce ICU mortality.

## Introduction

The intensive care unit (ICU) is a separate, specially staffed, and equipped hospital unit dedicated to the observation, care, and treatment of patients with life-threatening conditions^1–3^. Intensive care could reduce mortality rates by 15–60% when well-equipped and staffed with intensivists^2^. Studies in resource-rich settings showed that the physician, nurse, pharmacist, respiratory or physical therapist, and dietician staffing models in use affected the outcomes of critically ill patients^4^. However, intensive care specialists are unavailable in resource-limited settings, and the ICUs are served by anesthetic officers in collaboration with surgeons, internists, and pediatricians^5^. In sub-Saharan Africa, ICUs have varying qualities and quantities of infrastructure, mainly determined by the country's economic level^6^. In Ethiopia, ICU equipment and supplies are also limited, in addition to ICU staff shortages, causing a lag in the quality of ICU services relative to the standard^7–9^.

The mortality rate in critical care units worldwide ranges from 9 to 61%^10^. Different studies reported that the ICU mortality rate varied across the globe. North America (9.3%), Oceania (10.3%), Europe (18.7%), South America (21.7%), and the Middle East (26.2%) have relatively low rates of ICU mortality^11^. However, the ICU mortality rate in Africa is high, ranging from 32.9 to 54%^12,13^. And the ICU mortality rate in Ethiopia fluctuates between 38.7 and 50.4%^9,14^. This makes it one of Africa's countries with the highest ICU mortality^7^.

Knowing the magnitude of medical intensive care unit mortality is very important for interventions in the health facilities of the Amhara region. Therefore, the current study is designed to assess the magnitude of mortality and its predictors among patients admitted to the ICU at Tibebe Ghion Specialized Hospital.

## Methods

### Study design, period, and setting

A retrospective cross-sectional study was conducted from February 24th, 2019, to January 24th, 2021, at Tibebe Ghion Specialized Hospital, which is 565 km away from Addis Ababa, the capital city of Ethiopia, in the North West direction. This hospital has six wards, two intensive care units (pediatric and adult), and 452 beds. The adult ICU had nine beds, six functioning mechanical ventilators, and one bedside ultrasound. This unit is staffed with two anesthesiologists, internal medicine specialists, sub-specialists, trained nurses, medical and surgical residents. It gives service to critically ill medical, surgical and gynecology, and obstetrics patients admitted from the emergency department, wards, operating rooms, and from referrals. The hospital has an average of 24 patients’ ICU admission in a month.

### Eligibility criteria

Clinically diagnosed and critically ill patients older than fifteen years and admitted to the adult ICU units were included. While patients whose charts were missing or incomplete or whose COVID-19 test results became positive and transferred to the COVID-19 treatment center were excluded from the study.

### Sample size

In this study, 648 patients were admitted in the last two years. All of the patient charts were reviewed. However, 568 critically ill patients who fulfilled the eligibility criteria were included.

### Study variables

#### Dependent variable

ICU mortality (No = 0, Yes = 1).

#### Independent variables

##### Sociodemographic characteristics

Age, sex, residence, and years of admission.

##### ICU admission-related characteristics

Source of admission, admission category, admission diagnosis, admission outcome, cause of death, length of stay in ICU, mechanical ventilation used, and the mental status during admission.

### Data collection tools and procedures

Data from ICU medical records were collected by trained ICU nurses using pretested structured data retrieval checklist. Regular check-up of data completeness and consistency was made daily.

### Data processing and analysis

Data were entered into Epi-data version 3.1 and analyzed using R version 4.0 software. Descriptive statistics were computed. Bivariate and multivariable logistic regression analyses were employed to assess the association between the exploratory variables and ICU mortality. A *p*-value of less than 0.2 in the bivariate analysis was considered for variables to be included in the multivariable logistic regression analysis. Variables with a *p*-value < 0.05 (AOR at 95%CI) on multivariable analysis were considered statistically significant predictors of ICU mortality.

### Ethical consideration and consent to participate

Ethical clearance was obtained from the institutional review board of Bahir Dar University with ethical approval number: 0034/2021. All the methods were carried out in accordance with relevant guidelines and regulations. All the experimental protocols were approved by an institutional review board of Bahir Dar University, and informed consent was obtained from the legal guardian (hospital director) as the study was conducted through a review of patient charts. Accordingly, a support letter was obtained from the Department of Internal Medicine and the chief clinical director of the hospital for permission to conduct the study and access patient charts from the Archive. Confidentiality of the patient's charts was kept secure, in which patients’ addresses and other identifications (Card No) were removed before analysis.

## Results

### Demographic characteristics among patients admitted to ICU

Six hundred forty-eight patients were admitted over two years period. Of these, 568 (88%) patients' charts had complete data. The median (± IQR) age of the respondents was 45 (± 33) years. More than one-third of the patients were in the age groups of 21–40 years. Nearly 59% of the patients were males and were admitted in 2020. Half of the admitted patients came from rural areas **(**Table 1**)**.

**Table 1:** Socio-demographic characteristics of patients admitted to the ICU of TGSH, 2021.

Variable	Category	Frequency	Percent
Age group (in years)	< 20	65	11.4
	21–40	202	35.6
	41–60	185	32.6
	61–80	102	18.0
	> 80	14	2.5
Sex	Male	332	58.5
	Female	236	41.5
Residence	Rural	282	49.6
	Urban	286	50.4
Year of admission	2019	234	41.2
	2020	334	58.8

### The intensive care unit admission related characteristics

About 74% of patients were admitted from the emergency department,, followed by the operation theatre (12.3%) and surgical ward (9.7%). Nearly 66% of the admitted patients were medical patients. Of this, infectious diseases (septic shock, pneumonia, and ARDS) were the commonest admission and causes of death. Near 1/3rd of patients were ventilated (Table 2).

**Table 2:** The ICU admission-related characteristics of patients admitted to TGSH, 2021.

Variable	Category	Frequency	Percent
Source of admission	Emergency department	422	74.3
	Medical ward	18	3.2
	Surgical ward	55	9.7
	Operation theater	70	12.3
	Gynecology/obstetrics	3	0.5
Admission category	Medical patient	371	65.3
	Surgical patient	191	33.6
	Gynecology/obstetrics	6	1.1
Admission diagnosis	Myocardial infarction	49	8.6
	Congestive heart failure	15	2.6
	Septic shock	61	10.7
	Pneumonia	41	7.2
	Acute respiratory distress syndrome (ARDS)	32	5.6
	Pulmonary thromboembolism	14	2.5
	Diabetic ketoacidosis	13	2.3
	Stroke	34	6.0
	Head trauma	96	16.9
	General Surgical	92	16.2
	Others*	121	21.4
ICU admission outcome	Improved	400	70.4
	Died	168	29.6
Cause of death	Myocardial infarction	5	3.0
	Congestive heart failure	4	2.4
	Septic shock	32	19.1
	Pneumonia	17	10.1
	Acute respiratory distress syndrome (ARDS)	13	7.7
	Diabetic ketoacidosis	2	1.2
	Stroke	11	6.5
	Head trauma	30	17.9
	Others*	54	32.1
Length of stay (days)	< 5	366	64.4
	5–10	122	21.5
	11–15	31	5.5
	16–20	28	4.9
	> 20	21	3.7
Glasgow coma scale	Conscious	72	12.7
	Confused	281	49.5
	Lethargic	122	21.5
	Comatose	93	16.4
Mechanical ventilation required at admission	Yes	185	32.6
	No	383	67.4

*Tetanus, GBS, Thyroid storm, Malignancy, Gastro intestinal bleeding, post-surgery, and poisoning).

### Trends of ICU admission and its outcomes

**Figure 1:**
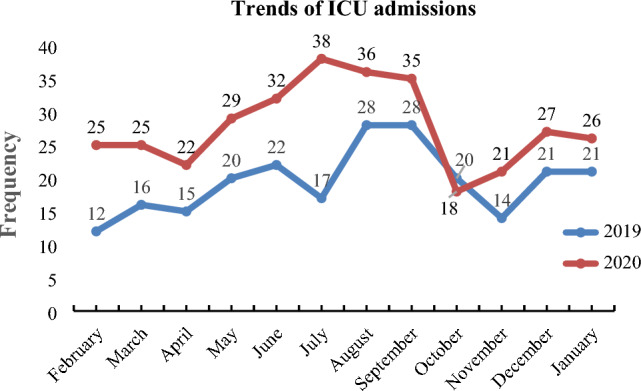
Trends of ICU admissions among patients admitted to TGSH, 2021.

In this study, 334 and 224 patients were admitted to the ICU in 2020 and 2019, respectively. The peak admission was observed in July 2020. In contrast, the lowest admission was seen in February 2019 (Fig. 1).

**Figure 2:**
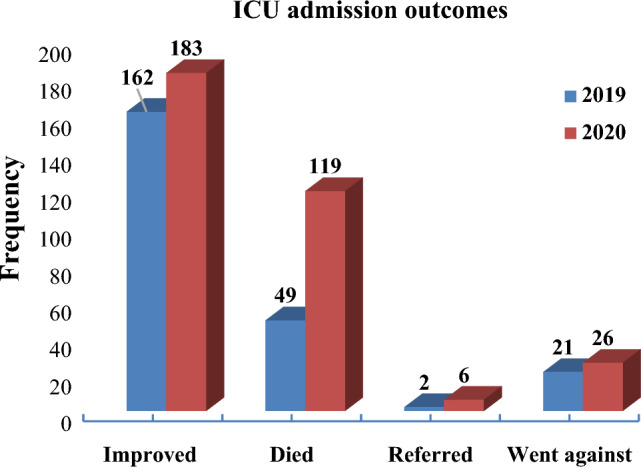
ICU admission outcomes among patients admitted to TGSH, 2021.

Regarding, the ICU admission outcomes, 20.9% (49/234) of the admitted patients died in 2019. While 35.6% (119/334) of the admitted patients died in 2020 (Fig. 2).

### The magnitude of ICU Mortality

The magnitude of death among patients admitted to ICU was 29.6% (95% CI: 26, 33).

### Factors associated with ICU mortality

Logistic regression analysis was conducted to identify independent predictors of mortality among patients admitted to the intensive care unit. Five predictor variables having a p-value < 0.2 at bivariate regression analysis were taken into a multivariable logistic regression analysis to see associations between dependent and independent variables. On the multivariable logistic regression model, four variables, namely year of admission, Glasgow comma scale, length of stay, and mechanical ventilation required at admission, were significantly associated with ICU mortality at a *p*-value of 0.05.

Accordingly, for those patients who were admitted in 2020 to the ICU, the odds of ICU mortality were about 49% (AOR = 0.51; 95% CI: 0.31, 0.85) less compared to patients who were admitted in 2019.

For those patients who had altered mentation, the odds of ICU mortality were 13.5 (AOR = 13.44; 95% CI: 5.77, 31.27) times higher than those who had stable mentation. Similarly, for patients who required mechanical ventilation at admission, the odds of ICU mortality were about four (AOR = 4.11; 95% CI: 2.63, 6.43) times higher than those who did not require mechanical ventilation at admission.

Similarly, for those patients who stayed < 5 days in the ICU, the odds of ICU mortality were about 3.7 (AOR = 3.74; 95%CI: 2.31, 6.06) times higher compared to those who stayed five and more days (Table 3).

**Table 3:** Factors associated with ICU mortality at Tibebe Ghion specialized hospital northwest, Ethiopia, 2021.

Variable	Category	ICU Mortality		COR (95%CI)	AOR (95%CI)	P-value
		Yes	No			
Year of admission	2019^*R*^	49	185	1.00	1.00	0.01
	2020	119	215	**2.09 (1.42, 3.07)**	**0.51 (0.31, 0.85)**	
Mental status	Stable ^*R*^	7	175	1.00	1.00	0.0001
	Unstable	161	225	**17.89 (8.18, 39.1)**	**13.44 (5.77, 31.27)**	
MV required at admission	Yes	95	90	**4.48 (3.05, 6.59)**	**4.11 (2.63, 6.43)**	0.0001
	No ^*R*^	73	310	1.00	1.00	
Length of stay	< 5 days	177	135	**5.15 (3.36, 7.91)**	**3.74 (2.31, 6.06)**	0.0001
	> 5 days^*R*^	223	33	1.00	1.00	
Blood pressure status	Stable ^*R*^	169	17	1.00	1.00	0.587
	Unstable	231	151	**6.49 (3.79, 11.14)**	0.78 (0.32, 1.90)	

*R* reference category, *MV* Mechanical ventilation.

Significant values are in bold.

### Discussion

The outcomes of patients admitted to the intensive care unit depend on the clinical condition of the patient’s arrival, the level of training and experience of staff, the resources, infrastructure, and capacity of the ICU unit^1,9^.

This study identified the magnitude of mortality and the predictors among patients admitted to the adult ICU of Tibebe Ghion specialized hospital. The study revealed that 29.6% (95% CI: 26, 33) of the admitted patients have died in the ICU. This finding was consistent with an indigenous study from Mekele of 27%^15^. This finding was higher than the studies done in South Africa, 19.7%^16^, and Canada, 19%^17^. However, it was lower compared to the studies from Gondar: 38.7%^9^, Addis Ababa 39%^14^, Jimma 50.4%^18^, Nigeria 34.6%^13^, and Tanzania 41.1%^6^. This discrepancy might be due to a lack of necessary medical equipment (an ABG analyzer machine, a portable dialysis machine, and a portable x-ray service), infrastructure, and training. In addition, the lack of a high-dependency unit in the study area and the fact that TGSH ICU is still in the new establishment might be contributing factors to the higher rate of ICU mortality. The other possible justification for the discrepancy might be differences in sample size, level of ICU care, availability of medical supplies, and stratification of skilled staff. However, compared to other study areas in Ethiopia and other African countries mentioned above, the mortality rate is lower. It may be justified by ongoing improvements in ICU services and training.

According to this study, patients admitted in 2020 had a 49% lower mortality rate than those admitted in 2019 (AOR = 0.51: 0.31, 0.85). As the first confirmed COVID-19 case was identified in Ethiopia on March 13, 2020, this low risk of death may be attributed to undiagnosed COVID-19 admissions as a result of the lack of COVID-19 screening tests during the early pandemic. However, after hearing about the COVID-19 instances, the majority of Ethiopia's health facilities were prepared to handle a crisis^19^. As a result, patients who are brought to an ICU in 2020 may benefit from high-quality care and show signs of recovery.

Patients who stayed in ICU for less than 5 days were five times more likely to die than patients who stayed five or more days (AOR = 3.74: 2.31, 6.06), which is analogous to the study finding in Uganda^20^. However, the current finding varied from the study conducted in Hosanna; the length of ICU stay was more than 14 days, with ICU mortality^21^. The majority of the patients in the current study were elderly, with a mean age of 44.5 (± SD, 19.1) as opposed to study participants in Hossana (mean = 31.27 (± SD, 14.019)), which may be the likely explanation. Similar to this, in the current study 87.3%, of patients brought to the ICU were unconsciously compared to Hossana's study result (59.6%). While small intestinal obstruction (15.4%), brain injury (13.9%), and shock (10.4%) were the top causes of admission in Hossana’s study, head injury (16.9%), general surgery (16.2%), and septic shock (10.7%) were the leading causes in our study. Besides, the shortage of functional mechanical ventilators delays and denies the admission of critically ill patients to the medical ICU^22^.

The current study reported that those patients who required mechanical ventilation at admission had fourfold higher odds of ICU mortality than those who didn’t require mechanical ventilation. This finding was in agreement with the study findings of Gondar^9^, Kenya^12^, the United States of America^23–25^, the United Kingdom^26^, and Singapore^27^. The possible explanation for this association could be related to mechanical ventilation initiated for patients with respiratory failure who are unable to protect the airway and have hemodynamic instability. Furthermore, patients requiring mechanical ventilation are more vulnerable to ventilator-associated pneumonia and other nosocomial infections^28,29^. The other possible explanation could be that patients who used mechanical ventilation might be at a critical stage and have organ failure; this leads to a poor prognosis for their health problem and may end in death.

This study revealed that patients admitted with an abnormal mental status were more likely to die than conscious patients. The conscious disturbance is connected to severe decompensated disease, cerebral hypoperfusion due to sepsis, blood loss, poisoning, and neurological disorders^30^. In addition, in this study, severe head injury secondary to bullet injury was the most common cause of abnormal mental status and mortality.

## Limitations of the study

Due to the nature of the study design, a retrospective study based on the ICU registries and charts, only limited data were retrieved. So the necessary variables, which help identify independent risk factors for the clinical outcomes of patients admitted to the ICU, were not collected. In addition, data related to physiological and laboratory variables necessary to calculate severity and prognostic scores such as sequential organ failure assessment (SOFA), Simplified Acute Physiology Score (SAPS), and Acute Physiology and Chronic Health, Disease Classification System (APACHE) to predict ICU mortality were not collected due to an inability to locate them in the available written chart and incomplete ICU registry books.

## Conclusion

The magnitude of ICU mortality was moderate. The leading cause of death was shock, followed by head injuries. Years of admission, altered mentation, mechanical ventilation required at admission, and days of stay in the ICU were the predictors for ICU mortality.

## Data Availability

All data generated or analyzed during this study are included in this published article. However, the data can also be accessed from the corresponding author upon request.

## Abbreviations

AOR: Adjusted odds ratio
ARDS: Acute respiratory distress syndrome
CI: Confidence interval
GBS: Guliam barie syndrome
ICU: Intensive care unit
MV: Mechanical ventilation
